# Quality of life in children and adolescents with beta thalassemia

**DOI:** 10.1192/j.eurpsy.2024.1400

**Published:** 2024-08-27

**Authors:** A. Tsagkou, E. Evangelou, E. Vlachou, A. Zartaloudi, E. Dousis, C. Dafogianni, M. Polikandrioti, I. Koutelekos

**Affiliations:** ^1^ Greek Health System; ^2^University of West Attica, Athens, Greece

## Abstract

**Introduction:**

Children and adolescents with thalassemia suffer from chronicity of the disease and its treatment, including transfusion dependence and complications of iron overload.

**Objectives:**

To investigate the quality of life of children and adolescents with Beta Thalassaemia.

**Methods:**

This study is a cross-sectional study conducted at the Greek public Children’s Hospital. PedsQL ™ 4.0 Generic Core Scale (Greek version) was used to evaluate HRQOL in 41 thalassemia patients aged between 5 and 18 years and in 41 healthy controls of the same age range. For the analysis, the Statistic Package (SPSS ver.24) was used. Using Spearman’s correlation coefficient, t-test and MannWhitney tests were used, while for variables with three or more levels the Anova and Kruskall-Wallis. In order to investigate the relationship between two quantitative variables, Spearman’s correlation coefficient was used, while the relationship between two qualitative variables was used to control x2. As a statistical significance level, α = 5% was defined.

**Results:**

Of the 41 children with beta Thalassemia who participated in the study, 48.8% (n = 20) were boys and 51.2% (n = 21) girls. The mean age of children was 10.02 ± 4.10 years. For healthy children who participated in the study 51.2% (n = 21) were boys while 48.8% (n = 20) were girls. The mean age of the children was 9.63 ± 3.77 years. Children with Beta Thalassaemia have a lower quality of life in Physical Health and Activity(<0,001), Emotional Health(0,031), School Activities(0,008), Psychosocial Health(0,014), and the overall PedsQL 4.0 (<0,001)questionnaire compared to healthy children. Children between the ages of 5 and 7 have higher levels of quality of life in physical health and activity than older children(<0,001). In addition, children aged 5 to 7 have higher quality of life and overall PedsQL 4.0 score than older children(0,033) Children receiving combination therapy show better quality of life than children receiving subcutaneous therapy (total PedsQL 4.0 <0,001).

**Conclusions:**

Children and adolescents in all five categories had a better quality of life, after improved iron chelating methods and other psychosocial interventions.

**Disclosure of Interest:**

None Declared

